# Linguistic bias and the hidden costs of science lost in translation

**DOI:** 10.1128/aem.02229-25

**Published:** 2025-12-18

**Authors:** Gemma Reguera

**Affiliations:** 1 Department of Microbiology, Michigan State University 3078, East Lansing, Michigan, USA

**Keywords:** equity, unconscious bias, linguistic bias, implicit bias, academic publishing

## Abstract

English has become the global language of science, but this dominance has a cost. Researchers who are not native English speakers face invisible hurdles: efforts to learn and use a second language, obstacles to research dissemination, and diminished professional visibility. These barriers do more than prevent access to opportunities. They cement unfair assumptions about scientific competence and preferentially amplify voices that are proficient, or perceived to be proficient, in the dominant language, shaping scientific discourse in narrow and exclusive ways. This editorial explores how linguistic bias sustains professional hierarchies and restricts scientific progress. It also highlights our journal’s initiatives to overcome language-based barriers in publishing and foster equitable participation in scientific exchange.

## EDITORIAL

Language is more than a tool for communication. It influences how we perceive and interact with others. Linguistic features such as accent, dialect, and word choice can trigger subjective perceptions of a person’s identity and cloud how we judge the skills and qualifications of one another. This phenomenon, known as linguistic bias, acts as an invisible barrier to scientific progress. It quietly upholds scientific hierarchies and stereotypes, and it effectively sidelines multilingual talent. At its root, linguistic bias is less about language itself and more about perceptions of proficiency and compliance with subjective standards. This bias finds a stronghold in academic publishing. Understanding how linguistic gatekeeping manifests allows us to challenge its impact and break down access barriers to multilingual talent.

## THE HISTORICAL PATH TOWARD LINGUISTIC HOMOGENIZATION

**Fig 1:**
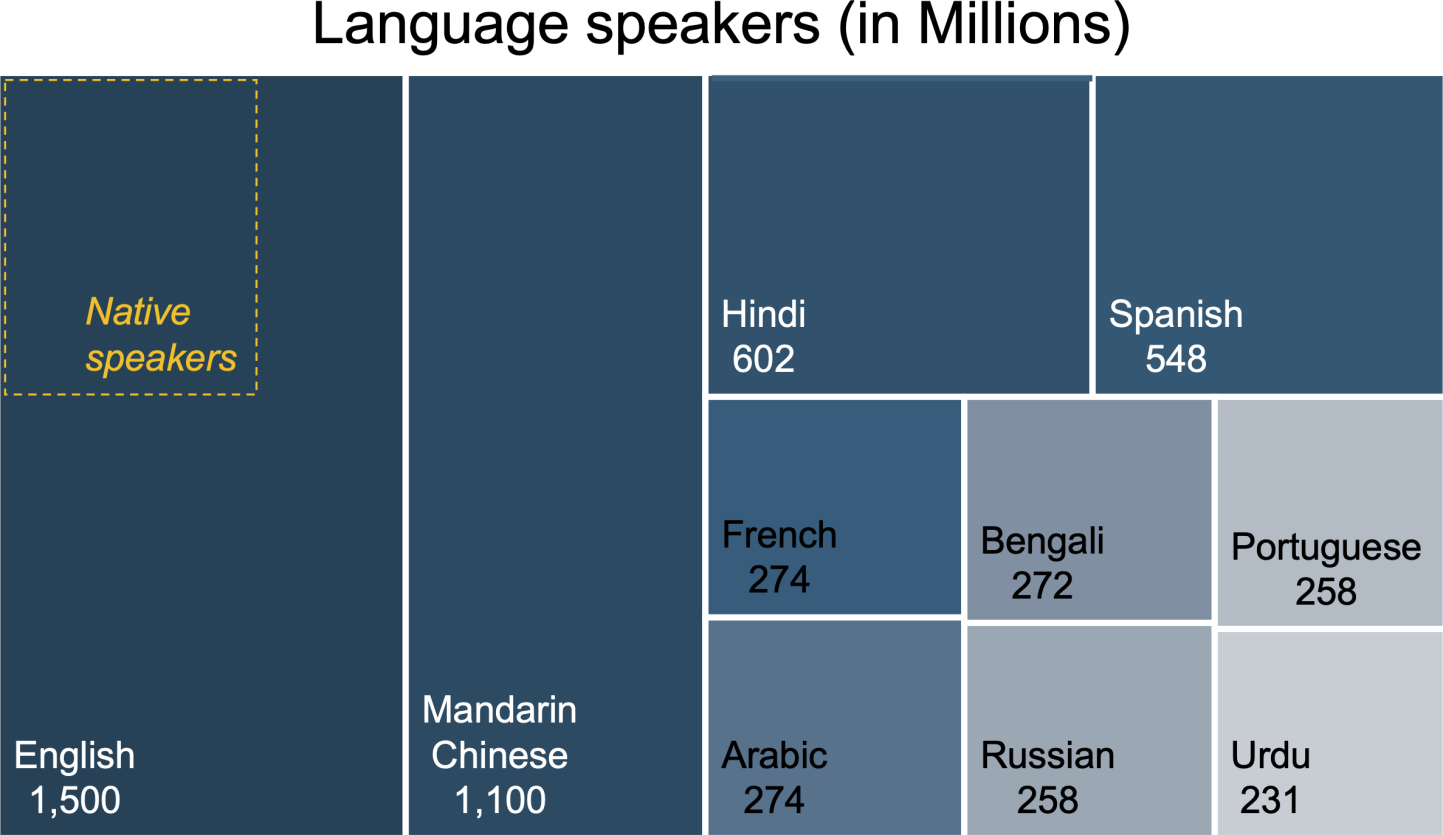
The 10 most spoken languages in the world. 2022 data on language speakers (in millions) from UNESCO World Atlas (1).

Languages use structured formats of symbols (e.g., oral, written, or gestured) for information exchange. The estimated 7,000 distinct languages spoken today are survivors of a linguistic history that once included more than 31,000 systems (1). But most of the planet’s population speaks only 10 of these languages, English being the most spoken of all (Fig. 1). Linguistic homogenization is rooted historically in centuries of British colonialism (2) and the more recent growth of the United States as an economic power (3). In a modern world of fast connectivity, English users can readily access and exchange information and make global connections, influencing politics, business, pop culture, science, and technology. Proficient speakers benefit from better-paid jobs, greater professional visibility, and more expansive networks of reach and opportunity. These incentives help explain why most English users are not native speakers (Fig. 1).

Science has also experienced linguistic homogenization. As the primary language for the dissemination of scientific knowledge, there is pressure on non-native scholars to communicate their scientific work in English (4). This immediately imposes the burden on multilingual scientists to present their work in the non-dominant language (5) and to conform to perceived linguistic standards of scientific discourse (6). As a result, a hierarchy of privilege based on English proficiency, or the perception of such (7), also exists that amplifies the most fluent voices and hinders scientific progress (8).

## LINGUISTIC BIAS IN ACADEMIC PUBLISHING

Language may be a tool for communication. But communication is a human experience and, as such, it is subjected to both conscious (explicit) and unconscious (implicit) biases (9). Unconscious bias is the unintended consequence of our brain’s tendency to process complex information via categorization, a process that reduces cognitive effort (“cognitive economy”) (10). This “shortcut” approach to information processing introduces assumptions that are conditioned by our emotions, life experiences, and social backgrounds. It also introduces assumptions about individuals based on their language.

Many aspects of linguistic bias are innate; others are acquired. Humans are conditioned to the unique speech patterns (11) and phonetic tonality (12) of their native language while in the womb and grow up as infants showing a preference for speakers of their native language (13). Research in the field shows just how deeply conditioned humans are to language. At 12 months, infants more readily take food from a native speaker than from a foreign speaker displaying similarly positive attitudes (14). By the age of 5 years, children favor friendships with native over foreign-accented speakers whom they can understand equally well (15). Linguistic bias is, therefore, deeply ingrained in the human brain and a strong conditioner of social perceptions and interactions. It also complex and plural and may manifest alone (7) or in combination with other biases, such as those associated with economic and geographical stereotypes (16, 17).

Biased assessments based on language style are well documented in science and are especially pervasive in academic publishing. In a modern culture of “publish or perish,” there is pressure on scientists to rapidly communicate their research to enhance their scholarly status and maximize the benefits of the modern academic reward structure (reviewed in reference 18). This competitive climate further disadvantages multilingual authors. A large randomized trial conducted in the journal *Functional Ecology* found significant differences in peer review outcomes based on linguistic perceptions (19). Peer ratings and editorial decisions favored authors perceived to be native speakers or to have native-like proficiency, and this bias was compounded by the economic status of the author’s country of origin. However, a double-anonymized (also known as double-masked or double-blind) peer review process to hide the authors’ identities equalized these ratings. These findings highlight the advantages associated with perceived English “nativeness” in academic publishing and the compounded effects of economic and geographical biases on peer review outcomes, as previously documented in other studies (20–22). In a separate study, slight deviations from standard English caused reviewers to perceive contributing authors as non-native speakers and rate the quality of their scholarly work lower than identical submissions with standard language patterns (23).

Linguistic bias may manifest in academic publishing in other ways too, such as positive associations between reviewers and authors who share the same country of affiliation (24) or those sharing the same primary language (25). Multi-tasking and deadlines, both common in academic publishing, are work stressors that favor categorical processing through our brain’s unconscious system (cognitive economy) (26), thus amplifying unconscious bias effects (27). As peer review workloads grow and reviewer fatigue sets in (18), multilingual authors become more vulnerable to language-based subjectivity.

## ACADEMIC PUBLISHING BEYOND WORDS

Simple solutions such as anonymizing author identities can help address, if only partially, the pervasive influence of linguistic bias in peer review (25). Open access tools assisted by generative artificial intelligence (AI), including chatbots such as ChatGPT, can assist multilingual authors to overcome some of the language barriers they face when disseminating their work. However, the use of AI tools has limitations. Users must know and abide by the journal’s legal and ethical policies. As stated by the Committee on Publication Ethics (COPE), “AI tools cannot meet the requirements for authorship as they cannot take responsibility for the submitted work” (28). Authors hold the ultimate responsibility for disclosing the use of AI tools and ensuring that the AI-generated content addresses conflicts of interest, adequately manages copyright or license agreements, and meets ethical standards of publication. Peer reviewers and editors also need to consider the confidential nature of the content under review and of their own evaluations when using AI tools. Submission of manuscript content, even if partial, to third-party AI platforms constitutes a breach of confidentiality for many journals. The American Society for Microbiology (ASM) has a generative AI policy that provides helpful guidelines to maximize the benefits of AI usage without compromising the integrity of the scientific record (29).

Programs that detect AI-generated language may also unfairly flag writings from multilingual authors as AI-generated content due to similarities in linguistic diversity and word choice (30). Like a human brain, AI tools apply the principles of cognitive economy to process information and make decisions expediently. And just as our own cognitive process, AI engines make assumptions and generalizations, sometimes biased, to optimally manage time and effort. Furthermore, these tools are trained on unvetted material and, therefore, they are susceptible to biases and inaccuracies. A case in point, when prompted with “How can you ask authors of a manuscript to address language deficiencies in a way that is not offensive?,” ChatGPT-generated text (31) suggested to do it “diplomatically and constructively so that it is helpful, professional, and non-offensive” but also recommended to do so “possibly with the help of a native speaker,” a deeply biased phrasing. When prompted to provide the source reference, ChatGPT referenced “similar phrasing” from a user of Academia StackExchange (32) who in fact argued against the referral to a “native” speaker (“Personally, I prefer to suggest that ‘the authors get editing help from someone with full professional proficiency in English’ rather than asking for ‘a native English speaker’*.*”), noting that “There are plenty of academics who are not native English speakers, and don’t have the same proficiency as native English speakers, but are still perfectly capable of high-quality academic writing.”

Self-awareness of linguistic bias, like other forms of unconscious bias, remains the most effective tool for scientists to mitigate its adverse effects. It starts with recognizing its pervasive influence (one cannot know what one doesn’t know). It then invites self-reflection to identify and challenge potentially subjective responses to linguistic aspects of works under review, such as grammar style and word choice. If the language does not preclude understanding, objective assessment of the scientific merit should be possible. Applying pre-established criteria and rubrics during the review, and not changing them mid-course, remains a staple of best practices toward peer review objectivity. For peer reviewers and editors, this means careful examination of technical merit and evidence-based assessments of scientific rigor and novelty. Editors also benefit from in-group discussions, interactions with peer reviewers, and coaching to collectively grow as a professional community focused on merit-based science.

As Editor in Chief of *Applied and Environmental Microbiology* (AEM), one of the flagship journals of ASM, I meet periodically with the team of editors and the 200+ members of our dedicated reviewing board to critically examine our peer review processes toward inclusive excellence. These exercises include active learning components where we examine contrasting examples of how anonymous peers and editors address concerns about language deficiencies. Below are examples from ASM’s archived reviews of an AEM submission that was flagged by three peer experts and one editor as having language deficiencies:

- The story is excellent […]; however, the **language needs to be improved** throughout.
- The authors are encouraged to **recruit the help of an expert** to substantially improve the paper’s English.
- The paper contains results of potentially serious pragmatic value but is **basically unreadable** in its current form.
- The **English usage is at best suboptimal**. Indeed, although the text is understandable in most cases, the **formulations are often awkward or incorrect**. In fact, there seems to be no sentence that does not need correction or should not be rewritten.[…] Please **show the manuscript to a native speaker** familiar with the topic of the study.
- The structure and language of the manuscript were **very poor**, making the study **extremely difficult to understand**.
- You must **rewrite** the text completely with **the help of someone with excellent English language skills**.

These comments clearly established that language editing could have elevated the presentation and value of this manuscript. But was the manuscript “unreadable?” It seems unlikely given the fact that three reviewers considered the science to be potentially transformative. Yet some recommended to “recruit the help of an expert,” “someone with excellent English language skills,” or “a native speaker” to “rewrite the text completely.” Furthermore, the manuscript was described by some reviewers as “basically unreadable,” “very poor,” “suboptimal,” and “extremely difficult to understand.” Such critiques further the marginalization of multilingual authors and could have been expressed more respectfully and constructively, as follows:

- “The authors may consider revising the manuscript for clarity and to better highlight the quality and significance of the research.”

Multilingual authors are all too familiar with the various ways in which peers communicate perceived linguistic deficiencies. Attempts to set and regulate the standards of academic English are all too common in social groups. This behavior, termed “verbal hygiene” (33), also impacts science. Scientists, like any other social group, tend to adopt a common linguistic style (what they perceive to be native or native-like) and regulate its use. It is in this context of perceived language standards and self-regulatory roles where linguistic bias manifests. Lost behind these behaviors is the fact that all scientists share the same understanding of the standards of scientific rigor (the scientific “method”) (34) and its value to support scientific inquiry and move the frontiers of knowledge beyond linguistic styles.

**Fig 2:**
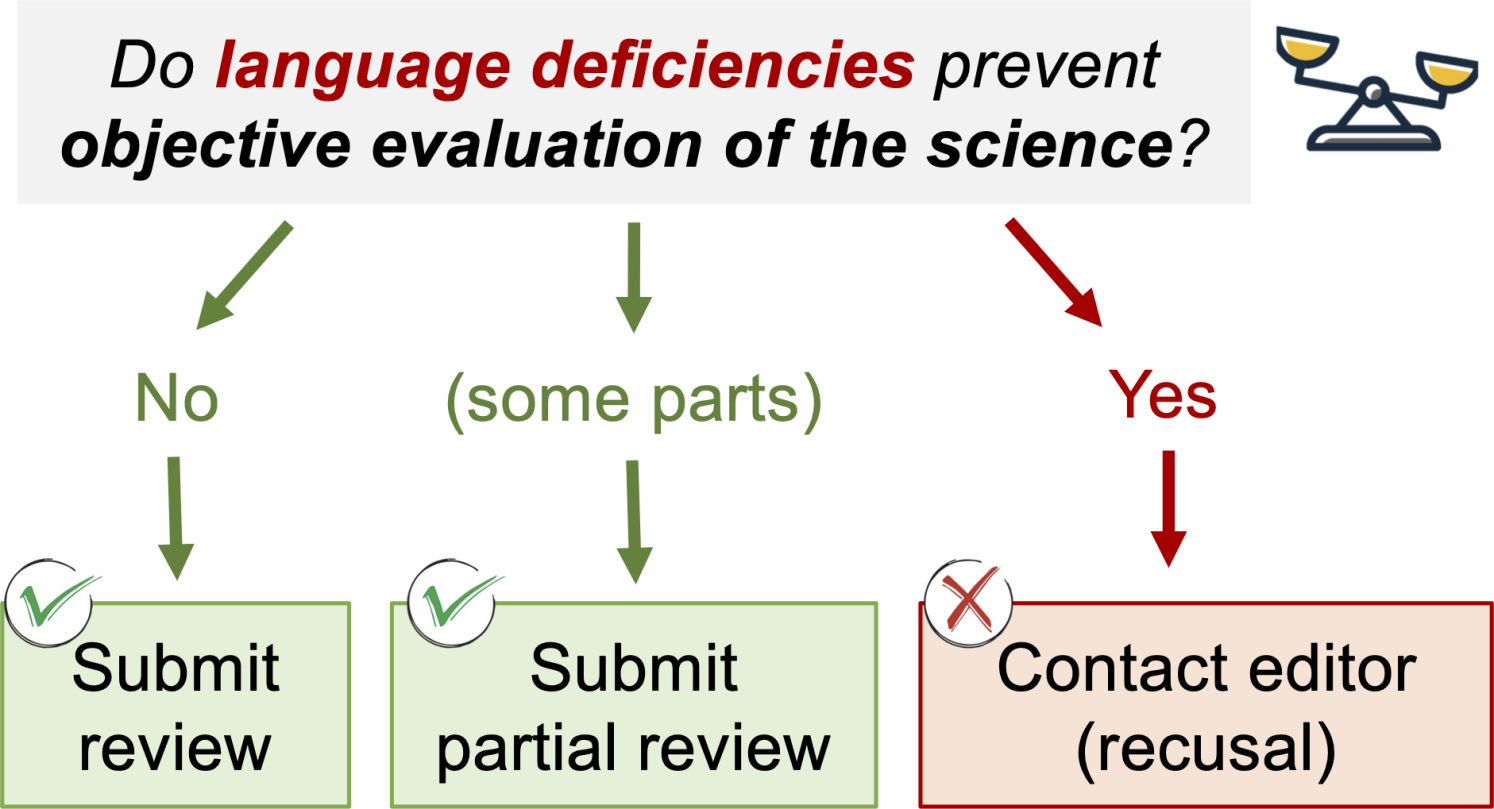
Flowchart for the management of linguistic perceptions during the peer evaluation of scientific papers.

What can scientists do to ensure impartiality under the often-opaque blanket of linguistic perceptions? Editor and reviewer training is essential to manage perceived linguistic barriers fairly, as it is disclosure of whether such barriers could cloud the objective evaluation of the scientific contribution. Reviewers should ask themselves, “Do language deficiencies prevent objective evaluation of the science?” and, if yes, recuse themselves from the review process (Fig. 2). Reviewers may also be guided to focus their evaluation on those parts of the manuscript that they can comfortably understand. Communication with the editor is essential to understand peer concerns and to maintain the integrity of the peer review process. Elevating such concerns to the Editor in Chief is encouraged to maintain rigor and consistency in all editorial transactions. These efforts have proven most valuable at AEM and could be replicated in other journals to better serve our global community of authors.

## A PATH TOWARD LINGUISTIC EQUITY

Impartiality, fairness, and respect are foundational values of a strong peer review and editorial process. Though intrinsic to human identity and shaped through lived experiences, scientists can proactively challenge linguistic assumptions to create spaces for multilingual opportunity and global engagement. Journals play a key role in supporting the voices of science. Double-anonymizing the peer review process is a necessary first step, as it is facilitating training programs that increase awareness of linguistic and associated biases. Our AEM workshops and guidelines to address linguistic bias are now used by other journals in the ASM family to ensure best practices in academic publishing and better serve our large, multilingual community of scientists. Advancing these initiatives empowers the academic publishing community to strengthen its commitment to integrity and rigor, ultimately unlocking access to a world of talent that would otherwise be lost in translation.
